# Serum TGF-*β*1 and CD14 Predicts Response to Anti-TNF-*α* Therapy in IBD

**DOI:** 10.1155/2023/1535484

**Published:** 2023-06-20

**Authors:** Stepan Coufal, Miloslav Kverka, Jakub Kreisinger, Tomas Thon, Filip Rob, Martin Kolar, Zuzana Reiss, Dagmar Schierova, Klara Kostovcikova, Radka Roubalova, Lukas Bajer, Zuzana Jackova, Martin Mihula, Pavel Drastich, Jana Tresnak Hercogova, Michaela Novakova, Martin Vasatko, Milan Lukas, Helena Tlaskalova-Hogenova, Zuzana Jiraskova Zakostelska

**Affiliations:** ^1^Laboratory of Cellular and Molecular Immunology, Institute of Microbiology of the Czech Academy of Sciences, Prague, Czech Republic; ^2^Laboratory of Animal Evolutionary Biology, Faculty of Science, Department of Zoology, Charles University, Prague, Czech Republic; ^3^Second Faculty of Medicine, University Hospital Bulovka, Dermatovenerology Department, Charles University, Prague, Czech Republic; ^4^ISCARE a.s., IBD Clinical and Research Centre, Prague, Czech Republic; ^5^Department of Gastroenterology and Hepatology, Institute for Clinical and Experimental Medicine, Prague, Czech Republic; ^6^Dermatology Prof. Hercogova, Center for Biological Therapy, Prague, Czech Republic; ^7^Institute of Medical Biochemistry and Laboratory Diagnostics, General University Hospital and First Faculty of Medicine, Charles University, Prague, Czech Republic

## Abstract

**Background:**

Tumor necrosis factor-alpha (TNF-*α*) agonists revolutionized therapeutic algorithms in inflammatory bowel disease (IBD) management. However, approximately every third IBD patient does not respond to this therapy in the long term, which delays efficient control of the intestinal inflammation.

**Methods:**

We analyzed the power of serum biomarkers to predict the failure of anti-TNF-*α*. We collected serum of 38 IBD patients at therapy prescription and 38 weeks later and analyzed them with relation to therapy response (no-, partial-, and full response). We used enzyme-linked immunosorbent assay to quantify 16 biomarkers related to gut barrier (intestinal fatty acid-binding protein, liver fatty acid-binding protein, trefoil factor 3, and interleukin (IL)-33), microbial translocation, immune system regulation (TNF-*α*, CD14, lipopolysaccharide-binding protein, mannan-binding lectin, IL-18, transforming growth factor-*β*1 (TGF-*β*1), osteoprotegerin (OPG), insulin-like growth factor 2 (IGF-2), endocrine-gland-derived vascular endothelial growth factor), and matrix metalloproteinase system (MMP-9, MMP-14, and tissue inhibitors of metalloproteinase-1).

**Results:**

We found that future full-responders have different biomarker profiles than non-responders, while partial-responders cannot be distinguished from either group. When future non-responders were compared to responders, their baseline contained significantly more TGF-*β*1, less CD14, and increased level of MMP-9, and concentration of these factors could predict non-responders with high accuracy (AUC = 0.938). Interestingly, during the 38 weeks, levels of MMP-9 decreased in all patients, irrespective of the outcome, while OPG, IGF-2, and TGF-*β*1 were higher in non-responders compared to full-responders both at the beginning and the end of the treatment.

**Conclusions:**

The TGF-*β*1 and CD14 can distinguish non-responders from responders. The changes in biomarker dynamics during the therapy suggest that growth factors (such as OPG, IGF-2, and TGF-*β*) are not markedly influenced by the treatment and that anti-TNF-*α* therapy decreases MMP-9 without influencing the treatment outcome.

## 1. Introduction

Inflammatory bowel diseases (IBDs), i.e., Crohn's disease (CD) and ulcerative colitis (UC), are severe chronic inflammatory illnesses of the gastrointestinal tract. IBD became a worldwide disease with constantly increasing incidence in Western and newly industrialized countries [1]. Alarming is the increasing onset of IBD in children and young adults [2]. Rather than being a single disease or two diseases (CD and UC), IBD is increasingly understood as a multifactorial disorder that is initiated and exacerbated by influences such as genetic susceptibility, environmental factors, aberrant immune response, and alteration in the intestinal microbiota [3].

In IBD therapy, the main focus is to induce and then maintain remission. Nowadays, biologic therapy, especially those targeting tumor necrosis factor-alpha (TNF-*α*), such as infliximab and adalimumab, successfully prevail in the management of IBD. Infliximab is a chimeric IgG1 monoclonal antibody that binds to soluble and membrane-bound TNF-*α*. Adalimumab is a fully human IgG1 isotype monoclonal antibody that binds both soluble and membrane-bound TNF-*α*. Each of these TNF-*α* inhibitors has a different structure, so the overall mechanism of its immunological action is different, and each has different efficacy and causes different side effects. In spite of the undeniable efficacy of anti-TNF-*α* therapy, almost 20%–40% of patients are primary non-responders, who fail to respond to TNF-*α* inhibitors within 8–12 weeks of initiating therapy. In another 20%–40% of patients, the response diminishes or fails within 1 year after therapy initiation [4–7]. The third reason for discontinuation of therapy is adverse drug reactions, such as tuberculosis, recurrent severe oropharyngeal infections, and paradoxical adverse skin manifestation, e.g., psoriasiform dermatitis [8–10].

Predictors of anti-TNF-*α* therapy response in IBD patients that might avoid unnecessary treatments are intensively studied but are often controversial due to different experimental conditions and small numbers of patients in study cohorts. In general, predictors of anti-TNF-*α* response can be divided into three groups: patient-related factors, disease-related factors, and treatment-related factors. Patient- and disease-related factors include independent aspects that exist prior to initiation of anti-TNF-*α* treatment, while therapy-related factors could be monitored after initiation up to 1 year of duration of anti-TNF-*α* therapy [11]. In both UC and CD, factors associated with a good response include younger age at diagnosis, concomitant use of an immunomodulator, and inexperience with previous anti-TNF-*α* therapy, shorter disease duration, elevated C-reactive protein (CRP) [12–15]. Additionally, in CD patients, it is an absence of previous surgery, and in UC patients, a lower hemoglobin level [16, 17]. On the contrary, predictive factor for primary non-response is mostly high BMI and severe course of disease [18, 19]. Genetic profiling suggested several genes associated with responsiveness to infliximab in UC patients. All those genes, namely osteoprotegerin (OPG), stanniocalcin-1, prostaglandin-endoperoxide synthase 2, interleukin (IL) 13 receptor alpha 2, and IL-11, are important in the adaptive immune response, inflammation, and TNF signaling pathways [20]. Recently, polymorphisms in genes involved in activating NF-*κ*B through the Toll-like receptor (TLR) signaling pathways, genes regulating TNF-*α* signaling, and cytokines regulated by NF-*κ*B are important predictors for the response to anti-TNF-*α* therapy among patients with IBD [21–23]. Nowadays, all well-established methodologies are usually evaluated from intestinal biopsies, which is invasive operation for patients accompanied by a risk of perforation. Searching for serum biomarkers as a non-invasive and convenient approach has become a focal point in IBD research.

Up to date, there is a limited number of studies. Reinisch et al. [15] conducted multicenter, randomized, placebo-controlled study where they showed that a serum CRP level of 0.5 mg/dl at week 14 in responders to infliximab induction therapy was a good indicator of response to infliximab therapy. Since the imbalance between matrix metalloproteinases (MMPs) and tissue inhibitors of metalloproteinases (TIMPs) plays an important role in the remodelation of extracellular matrix of intestinal wall during IBD, Carbon et al. [24] showed that reduced levels of serum TIMP-2 but not TIMP-1, MMP-8, MMP-9 at 14 weeks from baseline predicted good response to anti-TNF-*α* therapy.

As TNF-*α* inhibitors are frequently used in IBD patients, an accurate disease assessment and prediction of therapeutic response have become critical challenges in clinical practice. Genetic profiling, metabolomics, and microbiome studies are currently needed to uncover how to predict the response to anti-TNF-*α* therapy. For this reason, we conducted this pilot study, where we searched for non-invasive serum biomarkers that could be able to predict the therapy response. The selection of the molecules was based on our previous work, where we identified several molecules in IBD patients associated with anti-TNF-*α* therapy (OPG, CD14, endocrine-gland-derived vascular endothelial growth factor (EG-VEGF)), disease activity (transforming growth factor-*β* (TGF-*β*), trefoil factor 3 (TFF-3), MMP-9, lipopolysaccharide (LPS)-binding protein (LBP), CD14, mannan-binding lectin (MBL), insulin-like growth factor 2 (IGF-2), MMP-14) as well as with discrimination of IBD patients (MMP-9, MMP-14, MBL, TFF-3, EG-VEGF, TIMP-1, IGF-2) from healthy individuals [25]. To cover the main steps in the IBD pathogenesis for therapy response analysis, we also included markers of gut barrier damage (intestinal fatty acid-binding protein (I-FABP), liver fatty acid-binding protein (L-FABP)) and members of the IL-1 family (IL-18, IL-33), which are known to play a role in the barrier immunity as well as in the etiology of several inflammatory disorders, including IBD [26, 27].

## 2. Material and Methods

### 2.1. Study Cohorts

All consecutive patients naive to biological therapy between 18 and 65 years of age with CD or UC in whom biological treatment with infliximab or adalimumab was indicated between November 2018 and December 2020 were considered for inclusion to the study. The patients had to pass routine pretreatment screening before introducing immunomodulatory treatment and provide informed consent with participation in the study. Patients with antibiotic treatment 3 months or less before the start of anti-TNF-*α* were excluded. Moreover, patients after proctocolectomy or subtotal colectomy, patients with stoma, and pregnant or breastfeeding women were excluded from participation in the study. Prevailing indication for biological treatment in a vast majority of prospective population was luminal disease activity failing to respond to conventional treatment with immunosuppressive agents. In total, 38 IBD patients were recruited at the IBD Clinical and Research Centre, Prague, Czech Republic. In this pilot prospective cohort study group, 26 patients had UC and 12 patients had CD. Of this cohort, 17 patients were treated with infliximab and 21 with adalimumab. All participants in our study were Caucasians. Patients were stratified according to the therapy response in groups with no response (NR), partial response (PR), and full response (FR). The clinical characteristics of the studied cohorts are shown in Table 1. The therapy responding cohort (validation cohort) was composed of long-term responding patients to anti-TNF-*α* therapy suffering from CD (infliximab N = 30; adalimumab N = 11) or UC (infliximab N = 14; adalimumab N = 2) was included to study the level of selected molecules after long term successful anti-TNF-*α* therapy. Sampling from this cohort was done on a cross-sectional basis. Subjects in this control group were defined as patients who (a) reached clinical and endoscopic remission 6 months after anti-TNF-*α* therapy initiation (b) were continuously on anti-TNF-*α* therapy for at least 24 months at the time of sampling (c) were in full clinical remission for ≥6 months prior to sampling. The sampling of these patients was carried out on the day of their regular check-up just before the scheduled biological treatment administration. Furthermore, we included serum samples obtained from healthy individuals (*N* = 46) (Table S1).

### 2.2. Evaluation of Disease Severity and Therapy Response

Patients were followed in regular intervals coincident with drug applications, including weeks 0, 2, 6, and then every 8 weeks for infliximab and weeks 0, 2, 8, and then every 6 weeks up to week 38 for adalimumab. At each visit, blood sample was taken for analysis of blood count and biochemistry, and stool sample was collected for measurement of fecal calprotectin (FC). Clinical disease activity was registered using the Harvey–Bradshaw index (HBI) for CD and partial Mayo score (pMayo) in patients with UC [28, 29]. HBI or pMayo were calculated by specialized IBD gastroenterologists during patients' visits at the outpatient department according to disease activity reflected by patient outcomes and physician's assessment. In order to avoid interobserver variability in this study, overall response to therapy was evaluated using physician's global assessment (PGA) score by a single evaluator. Primary aim was the evaluation of response to therapy at week 38 based on laboratory and clinical markers of activity combined. Normalization of laboratory markers included CRP of <5 mg/l and FC < 150 *µ*g/g. Normalization of clinical disease activity included HBI < 5 for CD or pMayo < 2 for UC. Patients were evaluated as FR at week 38 when normalization of both clinical disease activity index and laboratory markers of inflammation occurred. PR included individuals with normalization of only one of either clinical or laboratory markers or at least 50% decrease in both clinical and laboratory markers [30, 31]. NR did not fulfill any of these criteria.

### 2.3. Serum Biomarkers Detection

Serum samples from IBD patients were obtained at the baseline and week 38 of therapy, samples from healthy controls and validation cohorts were collected at a single time point. Serum was aliquoted and stored at −80°C until analyses. Analyzed biomarkers associated with gut barrier function and inflammatory response were quantified by commercial enzyme-linked immunosorbent assay (ELISA) in serum (Table 2). Due to the limited amount of samples, we were not able to measure TNF-*α* levels in two PR patients and two FR patients.

### 2.4. Statistical Analysis

Non-parametric Kruskal–Wallis test with Dunn's multiple comparison and mixed-effects model analysis with Sidak's multiple comparison tests were used to compare multiple experimental groups (in one time point) and for longitudinal data analyses (week 0 and week 38). The Mann–Whitney test was used to compare two experimental groups, and Wilcoxon matched-pairs signed rank test was used to compare paired data inside one group. The data are presented as mean with standard deviation (if not stated otherwise). Differences were considered statistically significant at *p* ≤ 0.05 unless otherwise stated. GraphPad Prism statistical software (version 8.1.1, GraphPad Software, San Diego, CA, USA) was used for statistical analyses. Other analyses were performed on the R statistics platform (R Foundation for Statistical Computing, http://www.r-project.org) [32]. The relation of individual biomarkers to NR, FR, or PR was visualized by the nearest shrunken centroid method using the PAM package (ver. 1.56.1) [33]. Regression analysis compared the effect of each biomarker on Akaike information criterion (AIC) using the nnet package (ver. 7.3-12) [34]. Next, we performed both backward elimination and forward selection based on AIC to determine the best regression model to discriminate between the patients with no response and those with partial and full response. The composite receiver operating characteristic (ROC) curves were constructed and their area under the curve (AUC) was calculated using ROCR package (ver. 1.0-7) [35]. Hierarchical clustering using the Spearman distance metrics and heatmap construction was performed in the ComplexHeatmap (ver. 2.6.2) package for R [36]. Next, we explored variation in biomarker concentrations using principal component analysis (PCA) in package FactoMineR for R (ver. 2.4.) [37]. Systematic differences in biomarker profiles among patient groups were tested by redundancy analysis (RDA). To eliminate the effect of extreme values, concentrations of all biomarkers were square root transformed prior to PCA and RDA analyzes. The interconnection between the molecules was shown by the protein–protein network functional enrichment analysis based on coexpression, cooccurrence, experimentally determined, and both text mining and curated databases through the STRING database and STRING Consortium 2022 web source [38].

## 3. Results

### 3.1. Baseline Serum Biomarker Profile Distinguishes Future FR and NR to Anti-TNF-*α* Therapy

**Figure 1:**
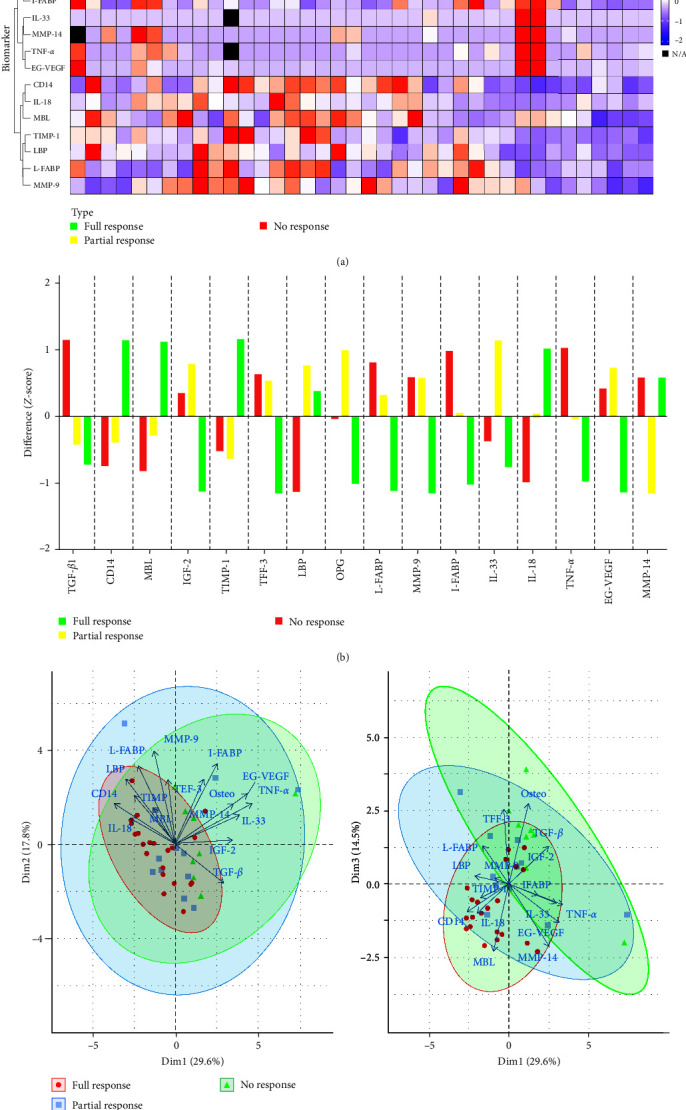
Patients who do not respond to biological therapy have a distinct serum biomarker profile at baseline, as measured by (a) hierarchical clustering heatmap, (b) nearest shrunken centroids classification, and (c) principal component analysis (PCA). PCA biplots show 1st and 2nd and 1st and 3rd PCA axis. Ordination space occupied by each sample group is highlighted by standard 95% ellipses.

Serum biomarker profiles at baseline distinguish patients with full response from those without any response 38 weeks later (Figure 1(a)). Interestingly, patients with partial response did not show any distinct clustering and were scattered throughout both clusters. This suggests that partial response being halfway between FR and NR in both clinical outcome and biomarker levels. Patients with no response have generally higher OPG, IGF-2, TFF-3, and TGF-*β*, and patients with full response have generally higher CD14, MBL, and TIMP-1 (Figure 1(b)). Interestingly, when the three outcomes were compared, levels in partial-responders (PR) followed non-responders (NR) in CD14, MBL, TFF-3, and TIMP-1 and full-responders (FR) in TGF-*β* (Figure 1(b)). The PCA has proven this distinct clustering of NR group compared to FR and PR along the first axis (positively saturated, especially by TNF-*α*, EG-VEGF, I-FABP, and IL-33) and partially also along the third axis (negatively saturated by OPG, TFF-3, and TGF-*β*, positively by MBL and MMP-14) (Figure 1(c)). These differences in biomarker profiles among experimental groups were statistically significant when compared by RDA (*F*_(2,38)_ = 2.585, *p* = 0.022). A simplified RDA focused only on differences between patients with no response and other patients (i.e., full and partial response) also showed a significant difference as well (*F*_(1,39)_ = 3.580, *p* = 0.017). Its explanatory power was comparable with original RDA testing differences among all three patient groups (*F*_(1,39)_ = 1.539, *p* = 0.222), suggesting that the main variation was associated with a therapy response or non-response irrespective of the response magnitude. This conclusion was supported by an RDA running on a data subset with excluded samples from NR group that did not detect any difference between PR and FR group (*F*_(1,31)_ = 1.507, *p* = 0.197). RDA biplot is depicted in Figure S1.

### 3.2. Anti-TNF-*α* Therapy Influences Serum Biomarker Dynamics Differently

**Figure 2:**
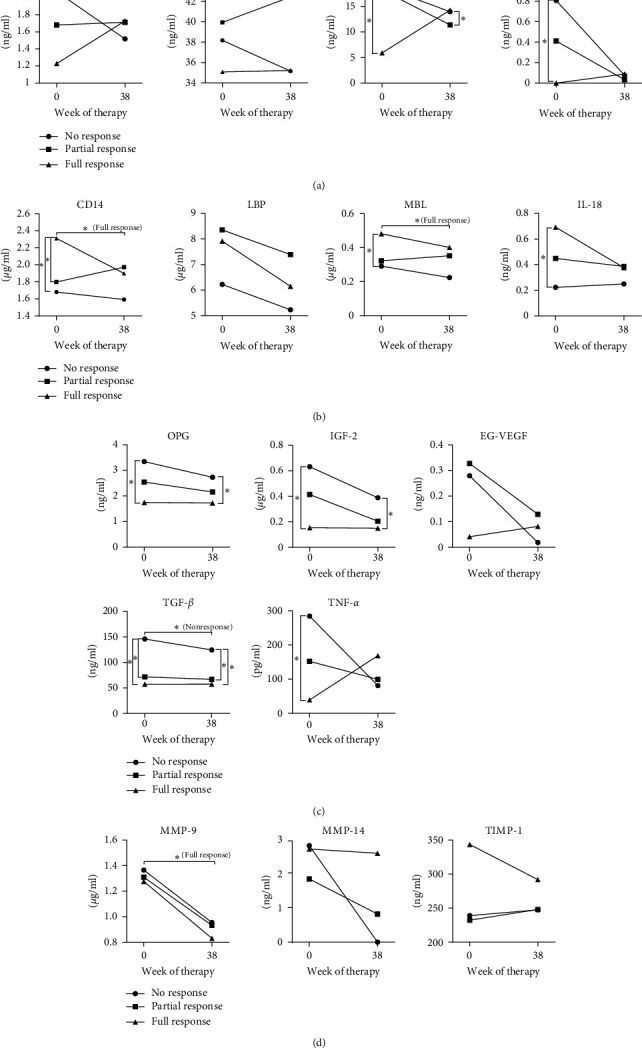
Changes in biomarker level during anti-TNF-*α* therapy: (a) molecules associated with gut barrier damage; (b) molecules involved in immune response associated with microbial translocation; (c) molecules regulating the immune response; (d) molecules of the matrix metalloproteinase system. The level of biomarkers is depicted as a mean of values; statistically significant differences between groups or between time points are marked with  ^*∗*^ ( ^*∗*^*p* < 0.05).

During the 38 weeks of therapy, dynamics of these biomarkers differed depending on the outcome. There was a general decrease of initially high levels of molecules associated with gut epithelium damage (I-FABP, L-FABP, TFF-3, and IL-33) in NR group (Figure 2(a)) and a similar decrease of molecules involved in the immune response associated with microbial translocation (e.g., CD14, MBL, and IL-18) in patients with full response (Figure 2(b)). And while growth factors involved in the regulation of immune response (e.g., OPG, IGF-2, and TGF-*β*) still showed significant differences between FR and NR groups (Figure 2(c)), levels of MMP-9 decreased in all groups during therapy in a similar degree (Figure 2(d)). This suggests that growth factors, such as OPG, IGF-2, and TGF-*β*, are not markedly influenced by the treatment and that anti-TNF-*α* therapy decreases MMP-9 without influencing the treatment outcome. TNF-*α*, originally well separated among the groups, evened out during the treatment. Taken together, patients who do not respond to the therapy have higher degree of gut epithelium damage with lower immune response to microbial translocation already at the baseline; thus, the failure of anti-TNF-*α* therapy may not be mediated by TNF-*α*-dependent mechanism.

### 3.3. Predictive Performance of Analyzed Biomarkers

**Figure 3:**
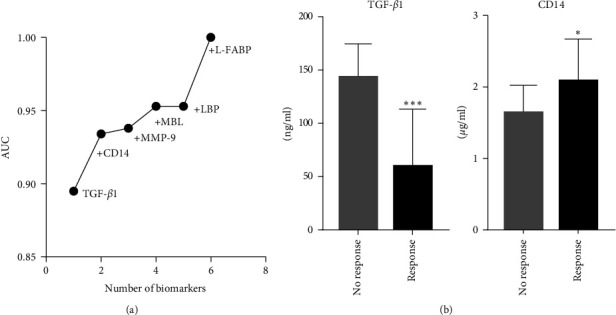
Diagnostic performance to predict therapy response: (a) relative importance of individual biomarkers in distinguishing NR from FR and PR group within the best model found by regression analysis; (b) quantitative plot of the two most efficient discriminating factors analyzed by Mann–Whitney test, the biomarker levels are depicted as mean ± SD of the values ( ^*∗*^*p* < 0.05;  ^*∗∗∗*^*p* < 0.001).

Next, we analyzed how the levels of these biomarkers may distinguish patients with no response from those with response (both full and partial) to anti-TNF-*α* therapy. First, we ranked all biomarkers according to their ability to distinguish these groups and found that TGF-*β*1, CD14, MMP-9, MBL, LBP, and L-FABP were relevant variables for this distinction (Table S2). We compared biomarker levels at baseline, finding that low TGF-*β*1 and high CD14 at the baseline have together high discrimination power for the anti-TNF-*α* therapy response at week 38 (AUC = 0.936) (Figure 3). The individual ROC analyses of the first three molecules in this model, including the calculated AUC, are depicted in Figure S2. To confirm our observation, we analyzed the serum level of TGF-*β*1, CD14, and MMP-9 also in samples obtained from CD and UC patients undergoing the successful long-term infliximab or adalimumab therapy determined by the PGA as described previously (Figure S3). We did not find any significant differences in the level of these molecules neither between CD and UC diagnoses nor the infliximab and adalimumab therapy in this cohort. Interestingly, the levels of these molecules found in PR and especially in FR at the week 38 were the most similar to the levels found in patients with successful long-term anti-TNF-*α* therapy.

**Figure 4:**
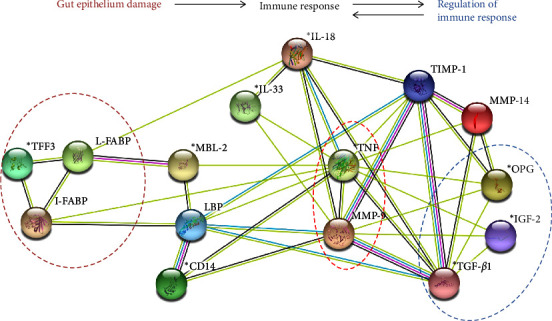
The network depicting the protein–protein interaction between selected biomarkers delineating the gut epithelium damage and inflammatory response. The interconnection biomarkers shown by protein–protein network functional enrichment analysis based on coexpression (black line), co-occurrence (dark-blue line), experimentally determined (pink line), and both text mining (yellow line) and curated databases (blue line). The statistically significant differences between NR and FR groups in biomarker level at the baseline are marked by  ^*∗*^. Network was produced through the STRING database and STRING Consortium 2022 web source. The dark-red dotted circle depicting the molecules associated with the damage of gut epithelium, the blue dotted circle depicting molecules associated with the regulation of immune response, and the red dotted circle showing the TNF-*α* and MMP-9 in the center of this protein–protein interaction network of molecules analyzed in IBD pathogenesis and therapy response.

To gain insight into the interactions between these molecules in the organism, we performed also the protein–protein interaction network functional enrichment analysis of selected biomarkers. This analysis delineates the possible pathway in the IBD pathogenesis showing the interconnection between gut epithelium damage (I-FABP, L-FABP, TFF-3), reflected by molecules recognizing microbial translocation (MBL, LBP, CD14) and subsequent immune response (IL-18, IL-33) with a central role of TNF-*α* and MMP-9. This model is accompanied by growth factors (IGF-2, TGF-*β*1, and OPG) acting here as regulators of immune response (Figure 4). Thus, this model enables complex perspective on the interaction between all molecules used in this study.

## 4. Discussion

The advent of anti-TNF-*α* agents as the first approved targeted therapy in the treatment of patients with IBD has made an important impact on existing therapeutic algorithms [39]. Anti-TNF-*α* therapy leads to mucosal healing, reduces hospitalization and surgery, and improves patients' quality of life [30]. In spite of this tremendous progress made in IBD therapy in recent years, approximately 30% of patients are primarily unresponsive to anti-TNF-*α* treatment and even among responders, up to 10% lose their response to the drug every year [40]. Thus, there is a need to establish predictive markers of response to identify the subgroup of IBD patients with a heightened probability of response that might avoid unnecessary treatment.

In our previous study, we used the broad-spectrum protein microarray analysis for screening the sera from IBD patients, including CD, UC, patients with primary sclerosing cholangitis (PSC), and PSC-IBD patients. This approach allowed us to analyze in total of 507 human proteins, including cytokines, chemokines, adipokines, growth factors, angiogenic factors, proteases, soluble receptors, and soluble adhesion molecules in one serum sample. Using this approach, we were able to establish a biomarker panel reflecting the main steps of the pathogenesis of different forms of IBD (CD, UC, PSC-IBD), including the screening of humoral and cellular adaptive immune responses against the gut commensal microbiota [25]. The molecules selected for this current pilot study are often found in the literature related to each other, as we documented by protein–protein interactions network functional enrichment analysis. This gave us a unique opportunity to combine current knowledge in the field to perform non-invasive analysis of molecules delineating the pathogenesis of IBD and find out suitable biomarker pattern for non-invasive assessment of the therapy response. Thus, in our study, we analyzed the power of serum biomarkers to predict the failure of anti-TNF-*α* therapy after 38 weeks. We used ELISA to quantify 16 serum biomarkers related to gut barrier function, microbial translocation, immune system regulation, and MMP system in IBD patients at the time of therapy prescription and 38 weeks later.

We found clear difference in biomarker profile between patients with no and full response prior to the anti-TNF-*α* therapy. The patients with only partial response were scattered through both clusters. This fact suggests that partial response occurs between no- and full response in both clinical outcomes and biomarker levels and the effect of redundancy in pro-inflammatory cytokines in PR group [41, 42]. Furthermore, patients with no response had increased level of molecules associated with gut epithelium damage (e.g., I-FABP, L-FABP, TFF-3, and IL-33) already prior to the therapy. We and others have previously described the intestinal and liver fatty acid-binding proteins as possible markers of gut epithelium damage and its importance for the early diagnosis of acute gastrointestinal diseases and necrotizing enterocolitis [26, 43–45]. The TFF-3 produced in the intestinal tract is associated with maintaining of the mucosal barrier integrity and promoting mucosal barrier restoration. The increase of TFF-3 levels found in non-responsive patients thus may represent a feedback mechanism triggered by gut epithelium damage, which in turn promotes mucosal barrier restoration [46, 47]. Expression of IL-33 is well established in the gastrointestinal tract, where IL-33 plays an essential role as an alarmin in the front-line mucosal immunity and in orchestrating the immune response following the gut barrier damage [48, 49]. As a pro-inflammatory signal, IL-33 binds to the surface receptor ST2, which enhances the activation of mast cells, Th2 T cells, and innate lymphoid cells type 2. In addition, IL-33 can also enhance the cytotoxic function of activated CD8^+^ T cells during ongoing Th1 immune responses [50]. IL-33 has been shown to be increased in active IBD patients, and blockade of IL-33 signaling alleviates active disease in experimental colitis [51–53]. Pastorelli et al. [54] reported that usage of anti-TNF-*α* therapy (infliximab) decreased circulating IL-33 in UC patients and that level of IL-33 was significantly increased and correlated with disease severity, suggesting that the IL-33/ST2 system plays an important role in IBD pathogenesis and it is modulated by anti-TNF-*α* therapy and may represent a specific marker for active UC [48, 49, 54]. Our results thus suggest that patient who will not respond to anti-TNF-*α* therapy in future have more extensive damage of gut epithelium than patients with full response in future already prior to the therapy. Surprisingly, these findings were not followed by an increased level of molecules associated with an immune response after microbial translocation (e.g., CD14, MBL, and IL-18) in these patients.

The main consequence of the gut barrier disruption is increased translocation of microbes, microbial toxins, and LPS from gut lumen, leading to activation of dendritic cells and macrophages followed by the production of pro-inflammatory cytokines resulting in inflammatory response. LPS-binding protein (LBP) is an important molecule in the recognition of LPS by TLR4, which in coordination with CD14 and MD2, leads to the activation of the NF-*κ*B signaling pathway and production of pro-inflammatory cytokines as TNF-*α*, IL-1*β*, and IL-6. This leads to infiltration of immune cells, increased tissue inflammation, and disruption of tissue homeostasis [55]. A dual, concentration-dependent, role of LBP has been reported. At low concentration, it enhances the LPS-induced activation of mononuclear cells, whereas at high concentration, LBP inhibits LPS-induced cellular stimulation due to LPS neutralization [56]. Richter et al. [57] supported this finding by demonstration that LBP improve wound healing of intestinal epithelial cell *in vivo* and it has a protective effect against LPS or Gram-negative bacterial infections at high concentration. While membrane CD14 is involved in LPS activation of CD14-positive cells (e.g., monocytes, macrophages, polymorphonuclear as well as non-myeloid cells such as B cells), CD14-negative cells (e.g., endothelial and epithelial cells) can be activated via soluble CD14. The soluble form is produced by protease-mediated shedding from the cell surface of leukocytes and also by hepatocytes. It has been shown that the level of soluble CD14 is elevated in different inflammatory conditions and correlates with IL-6 or CRP [58]. CD14 can also reduce the LPS-induced pro-inflammatory activities by competing with membrane-bound CD14 in a similar manner as described in LBP. Thus, soluble CD14 is suggested to has also a regulatory function in modulating of immune response to LPS [58, 59]. MBL is a soluble lectin acting as a pattern-recognition molecule, which acts as sense polysaccharide patterns on microbial surfaces and has the ability to activate the lectin complement pathway upon recognition of microorganisms, leading to opsonization and enhancement of phagocytosis by neutrophils, and modulate inflammation. MBL thus plays an important role in first-line defense against pathogens and in maintaining of intestinal homeostasis [60–63]. Several studies showed an association between MBL-deficiency and increased susceptibility to various infectious diseases [64]. Furthermore, MBL deficiency resulted in an excessive experimental colitis in response to mannose-expressing mild gut pathogens, suggesting that systemic MBL helps to prevent excessive inflammatory response following the penetration of normally mild pathogens through the disrupted intestinal epithelium [65]. Kovacs et al. [66] described an association between low MBL level and pediatric onset IBD. In children with CD was found a significantly higher frequency of *MBL2* gene variant responsible for MBL deficiency, suggesting the role of MBL in IBD pathogenesis [66, 67]. All these findings stress the importance of LBP, CD14, and MBL and their level in the immune response outcome associated with microbial translocation, which is in agreement with our findings from FR group. Similarly as IL-33, IL-18 belongs to IL-1 cytokine family. It is produced as a precursor molecule pro-IL-18, by a variety of cell types, including epithelial cells, myeloid cells, and lymphocytes. To become a biologically active molecule, it needs to be cleaved by caspase-1 after inflammasome formation. Its effector functions include stimulation of IFN-*γ* production, stimulation of Th1 T cell differentiation, and priming of NK-cell cytotoxicity [48, 68, 69]. Nowarski et al. [70] showed that IL-18 equilibrium controls barrier function in colitis and that overexpression of IL-18 leads to the breakdown of barrier integrity. Using *in vivo* model, they found that colitis severity was controlled at the level of IL-18 signaling and the role of goblet cell dysfunction in the IBD pathogenesis [70]. Recent studies suggest a dual role of IL-18 in colitis through the regulation of the function and quantity of goblet cells. Pretreatment with IL-18 reduced inflammatory infiltration and increased the MUC2 and TFF-3 production in mice with dextran sulfate sodium-induced colitis. By contrast, IL-18 treatment at a later stage of the disease enhanced inflammatory infiltration and reduced Muc-2 expression, decreased the function and quantity of goblet cells, suggesting the anti-inflammatory effect of IL-18 at the early stage of colitis-induced inflammation [71]. Here, we can only speculate if the higher concentration of IL-18 at baseline of future responders was associated with future response to anti-TNF-*α* therapy and increased goblet cell function and TFF-3 production.

Monocytes stimulated with EG-VEGF, a member of the VEGF family, have elevated IL-12 and TNF-*α* production, while the production of IL-10 was downregulated in response to LPS in humans [72]. This can lead to a decrease in the activation threshold of monocytes in intestinal wall which worsens the inflammation when intestinal barrier is compromised in the IBD. Interestingly, the increased levels of EG-VEGF we found in patients with no or only partial response to anti-TNF-*α* therapy already at the baseline. In patients with no future response, we found significantly higher level of TNF-*α* at the baseline as compared to patients with full response at week 38. The pro-inflammatory effect of TNF-*α* and its signaling plays a critical role in the pathogenesis of IBD, where in high concentration acts destructively through inflammatory response and gut barrier integrity breakdown via increasing the tight junction permeability [73, 74]. However, we observed a decrease in the level of TNF-*α* in patients with no response at week 38; the level of OPG was still significantly higher in these patients as compared to patients with full response. OPG belongs to the TNF-*α* receptor family and has an important role not only in the regulation of bone density but also in cell differentiation, survival, and death. Franchimot et al. [75] described the correlation of OPG and pro-inflammatory cytokines in IBD patients, suggesting that OPG production is influenced by cytokine milieu in chronic inflammation. Thus, high level of OPG at the week 38 may mirror persistent inflammatory response in these patients. Since the catabolism and growth impairment are well-known complications of IBD, Eivindson et al. [76] found a correlation between IGF-2 and IL-6 in IBD patients, suggesting an association between inflammation and the IGF system with involvement in muscle and bone catabolism in IBD pathogenesis. In our study, the IGF-2 level was significantly higher in patients with no response in both time points of monitoring as compared to patients with full response. However, we observed a significant decrease in TGF-*β*1 levels in patients with no response during the therapy course; the level was still significantly higher than in patients with full or partial response. Thus, our results suggest that the level of OPG, IGF-2, and TGF-*β*1 were not markedly influenced by the treatment. TGF-*β*1 is an important cytokine in the regulation of mucosal immune reaction contributing to maintaining of intestinal homeostasis [77]. In IBD patients were reported elevated levels of TGF-*β* showing the effort of organism to regulate the inflammatory conditions. In addition, it was shown that serum TGF-*β* levels increased in response to conventional IBD treatments, suggesting that TGF-*β* is required for the suppression of intestinal inflammation in active IBD patients [78, 79]. On the other hand, TGF-*β* plays a role in intestinal fibrosis and stricture formation in CD patients by increasing collagen production [80, 81]. The disturbance in the balance between synthesis and degradation of the extracellular matrix can result in typical features of IBD, such as ulcer formation, fibrosis, or organ destruction [82]. Therefore, we analyzed the proteins of MMP system, which are involved in the remodeling of extracellular matrix and connective tissue of the intestinal wall. We found decrease in the serum MMP-9 in all three groups at week 38, suggesting that anti-TNF-*α* therapy decreases MMP-9 level without influencing the treatment outcome. Our observation is in agreement with previous findings showing significantly higher levels of MMP-9 in IBD patients and that MMP-9 was proposed as a marker of mucosal damage and an independent predictor of both CD and UC [82–85]. On the other hand, we found only a slight decrease of MMP-14 in full-responders as compared to patients with partial or no response at week 38. Interestingly, it was shown that MMP-14 could act also as a negative regulator of inflammation during endotoxemia, suggesting regulatory function of MMP-14 [86, 87]. The next aim of our pilot study was to analyze how the level of these biomarkers could help in distinguishing of patients with future response and no response and thus avoid unnecessary treatment and early use of different therapy approaches. We found that low TGF-*β*1 and high CD14 at the baseline have the highest predictive power for the prediction of therapy response, discriminating patients with and without response with high accuracy. The protein–protein interaction network functional enrichment analysis on selected biomarkers supports our findings and delineates the involvement of these molecules in the model of gut barrier damage with subsequent inflammatory response resembling the IBD pathogenesis. This model shows the interconnection of the gut epithelium damage (here shown by I-FABP, L-FABP, and TFF-3) with sensors of microbial translocation (e.g., MBL, LBP, and CD14) and inflammatory response with the central role of TNF-*α* together with MMP-9. These results thus complement current findings that intestinal microbiota composition could be an important determinant of therapy response in IBD, and thus both baseline metagenomic and immune profiles are linked with therapy response [88].

Study subjects were limited to Caucasians; thus, our results may not be generalized for more diverse population. To exclude the variability caused by diagnosis or type of anti-TNF-*α* therapy, we validate our results also by independent measurement of TGF-*β*1, CD14, and MMP-9 in reference cohort composed of healthy individuals, patients suffering from CD or UC on long-term anti-TNF-*α* therapy (infliximab or adalimumab). We did not observe any significant differences in the levels of these molecules between CD and UC with infliximab or adalimumab. There are studies suggesting several other factors which could affect the therapy response, such as smoking, disease location, and severity. Previous study identified that smoking has a strong adverse effect on the response rate to anti-TNF-*α* therapy and maintaining of the response [89, 90]. With respect on the limited number of patients in our pilot study, we found that the NR group contained significantly higher amount of smokers as compared to both PR and FR groups. Another factor which could affect the therapy response is the disease location. Laharie et al. [91] showed that colonic involvement was the only predictive factor of therapy response in luminal CD. In our study, we did not observe this effect since the CD patients in FR group contain patients with colonic involvement, ileum involvement as well as patients with ileocolonic involvement. There were also differences in the therapy response between the types of drugs. Thorlund et al. [92] showed that infliximab was more effective than adalimumab in the induction of remission and mucosal healing of moderate to moderately severe UC at 8 weeks, but at the week 52, they became comparable in efficacy of maintenance. These results suggest that infliximab is more efficient than adalimumab in the short-term clinical response. Though, in our longitudinal observation, we were not able to observe a similar effect, in the validation cohort, there were more patients profiting from infliximab-induced remission.

## 5. Conclusion

In summary, we found that the biomarker profile mirror the therapeutic outcome already at the baseline. Therefore, it could be possible to identify the patients with a heightened probability of response prior to the therapy. Despite the pilot study design and the limited size of the study cohorts, our results are important for future research in the search for non-invasive biomarkers to predict the efficacy of a therapy success and may significantly improve the IBD patient management and decrease the disease and economic burden to the society. These results are bringing insight and highlighting the aspects of both immune and therapy response in non-invasive way for future work in this field.

## Figures and Tables

**Table 1:** Summary of anthropometric and clinical parameters from patients with IBD at baseline (prior therapy) and endpoint (week 38).

IBD (*N* = 38)						
Responsiveness to therapy	No response (NR group) (*N* = 7)		Partial response (PR group) (*N* = 11)		Full response (FR group) (*N* = 20)	
	Baseline	Week 38	Baseline	Week 38	Baseline	Week 38
CRP (mg/l)	2.6 (0.7, 4.3) (ab)	4.3 (2.4, 7.5) (a)	5.7 (1.2, 8.9) (ab)	3.2 (0.55, 5.8) (ab)	3.5 (1.3, 17.3) (a)	0.9 (0.5, 1.5) (b)

WBC	6.7 (5.2, 9.4) (ab)	8.2 (6.8, 11.1) (a)	7 (5.8, 9) (ab)	6 (5.1, 7.5) (ab)	7.1 (5.3, 10) (ab)	5.9 (5.1, 6.4) (b)

PLT	269 (251, 373) (ab)	363 (279, 435) (a)	343 (290.5, 370.5) (ab)	283 (216, 379.5) (ab)	281 (243.5, 370) (ab)	209.8 (254, 282.5) (b)

Ferritin (*µ*g/l)	31.59 (16.4, 55.6) (a)	30.9 (17.2, 55.9) (a)	40.6 (13.7, 67.2) (a)	16.8 (6.7, 89.8) (a)	58.3 (14.6, 126.4) (a)	51.7 (15.6, 74.7) (a)

Hb (*µ*g/l)	128 (127, 152) (a)	134 (130, 143) (a)	136 (128, 146.5) (a)	133 (111, 135) (a)	132.5 (112.8, 144.8) (a)	142.5 (132, 154.3) (a)

FC (*µ*g/g)	455 (162, 1,737) (ab)	764 (279, 976) (ab)	662 (274, 1,055) (ab)	426.5 (171.8, 1,596) (a)	479 (163, 858) (a)	40 (20, 137.5) (b)

BMI	19.84 (19.57, 22.15) (a)	22 (21.8, 30.4) (a)	23.1 (20.8, 26.7) (a)	20.8 (20, 24) (a)	22.3 (20.8, 23.9) (a)	23.4 (21, 25) (a)

Clinical score (pMayo)	8.0 (7.0, 9.0) (abc)	7.0 (7.0, 7.0) (ac)	6.5 (4.8, 7.5) (ab)	4.0 (2.0, 4.3) (c)	5.5 (2.5, 7.8) (ac)	0 (0, 0.8) (bc)

Clinical score (HBI)	6.0 (1.0, 7.5) (ac)	5.0 (4.5, 6.0) (ab)	3.0 (1.0, 5.5) (abc)	4.0 (1.0, 6.5) (abc)	1.5 (0, 3.8) (b)	0.5 (0, 1.0) (c)

Age (years)	33 (25, 44) (a)		29 (24, 47) (a)		29 (25, 41) (a)	
Gender (F, *N*)	7 (a)		9 (ab)		10 (b)	
Smoking (*N*)	4 (a)		1 (b)		1 (b)	
Diagnosis UC/CD (*N*)	2/5 (a)		3/8 (a)		7/13 (a)	
Infliximab/adalimumab (*N*)	1/6 (a)		4/7 (a)		12/8 (a)	
Immunosupressant agent (*N*)	2 (a)		7 (a)		11 (a)	
Disease duration (years)	6 (1, 10) (a)		3 (2, 8) (a)		4 (1, 12.75) (a)	
Age at diagnosis (years)	25 (17, 39) (a)		23 (21, 44) (a)		25.5 (20, 29.5) (a)	
Disease location						
CD_L1	0 (a)		4 (a)		6 (a)	
CD_L2	0 (a)		0 (a)		4 (a)	
CD_L3	5 (a)		1 (a)		6 (a)	
UC_E1	0 (a)		2 (a)		1 (a)	
UC_E2	0 (a)		2 (a)		1 (a)	
UC_E3	2 (a)		2 (a)		2 (a)	
Surgery history—resection (*N*)	1 (a)		2 (a)		1 (a)	

In total, we collected serum samples from 38 IBD patients. Medians are reported with the first and third quartiles in parentheses. N, number of participants; CD, Crohn's disease; UC, ulcerative colitis; HBI, Harvey–Bradshaw Index; pMayo, partial Mayo score; CRP, C-reactive protein; FC, fecal calprotectin; Hb, hemoglobin; PLT, platelet count; WBC, white blood cells; ns, not significant; Statistical differences between groups are depicted by letters at the level of *p* < 0.05. When same letter is present (e.g., a, a) in one row of the table, no statistical difference between group or time points is present. When two different letters are present (e.g., a, b) in the one row of the table, there is statistical difference between these observations at the level of *p* < 0.05.

**Table 2:** The list of quantified biomarkers in sera.

Biomarker	Abbreviation	Manufacturer	Cat. no.	Limit of detection
Endocrine-gland-derived vascular endothelial growth factor	EG-VEGF	R&D systems	DY1209	15.6 pg/ml
Insulin-like growth factor 2	IGF-2	R&D systems	DY292	23.4 pg/ml
Interleukin-18	IL-18	R&D systems	DY318-05	11.7 pg/ml
Interleukin-33	IL-33	R&D systems	DY3625B	23.4 pg/ml
Intestinal fatty acid-binding protein	I-FABP	R&D systems	DY3078	31.2 pg/ml
Mannan-binding lectin	MBL	R&D systems	DY2307	15.6 pg/ml
Matrix metalloproteinase 9	MMP-9	R&D systems	DY911	31.2 pg/ml
Matrix metalloproteinase 14	MMP-14	R&D systems	DY918	62.5 ng/ml
Osteoprotegerin	OPG	R&D systems	DY805	62.5 pg/ml
Lipopolysaccharide-binding protein	LBP	R&D systems	DY870	0.8 ng/ml
Liver fatty acid-binding protein	L-FABP	HyCult Biotech	HK404	102 pg/ml
Soluble CD14	CD14	R&D systems	DY383	62.5 pg/ml
Tissue inhibitor of metalloproteinase 1	TIMP-1	R&D systems	DY970	31.2 pg/ml
Transforming growth factor-*β*1	TGF-*β*1	R&D systems	DY240	31.2 pg/ml
Trefoil factor 3	TFF-3	R&D systems	DY4407	7.8 pg/ml
Tumor necrosis factor-alpha	TNF-*α*	R&D systems	DY210	15.6 pg/ml

## Data Availability

The data that support the findings of this study are available from the corresponding author upon request.

## Supplementary Materials

Supplementary Materials Table S1: Summary of anthropometric and clinical parameters from validation cohort composed of patients with IBD undergoing long-term successful anti-TNF-*α* therapy and healthy individuals. Table S2: The model performance information described by area under curve (AUC) and Akaike information criterion (AIC). Figure S1: RDA ordination. RDA biplot shows sample ordination along the two constrained RDA axes. Ordination space occupied by each sample group is highlighted by convex Hull polygons. Figure S2: The ROC analysis of TGF-*β*1, CD14, and MMP-9 analysis of ROC for individual markers with calculated area under curve (AUC) ( ^*∗*^*p* < 0.05;  ^*∗∗∗*^*p* < 0.001). Figure S3: The analyses of serum TGF-*β*1, CD14, and MMP-9 in therapy-responding cohorts. Analysis of the level of three main molecules based on our model in sera of IBD patients (CD, UC) with long-term anti-TNF-*α* therapy (infliximab or adalimumab) and healthy individuals (HC). Median is depicted.
