# Supplementary material for: Serum TGF-β1 and CD14 Predicts Response to Anti-TNF-α Therapy in IBD
Source: J Immunol Res. 2023 Jun 20;2023:1535484. doi: 10.1155/2023/1535484 (PMC10299888; doi:10.1155/2023/1535484)
Supplement: Supplementary Materials — Table S1: Summary of anthropometric and clinical parameters from validation cohort composed of patients with IBD undergoing long-term successful anti-TNF-α therapy and healthy individuals. Table S2: The model performance information described by area under curve (AUC) and Akaike information criterion (AIC). Figure S1: RDA ordination. RDA biplot shows sample ordination along the two constrained RDA axes. Ordination space occupied by each sample group is highlighted by convex Hull polygons. Figure S2: The ROC analysis of TGF-β1, CD14, and MMP-9 analysis of ROC for individual markers with calculated area under curve (AUC) ( ∗p < 0.05; ∗∗∗p < 0.001). Figure S3: The analyses of serum TGF-β1, CD14, and MMP-9 in therapy-responding cohorts. Analysis of the level of three main molecules based on our model in sera of IBD patients (CD, UC) with long-term anti-TNF-α therapy (infliximab or adalimumab) and healthy individuals (HC). Median is depicted. [file 1535484.f1.docx]

Article title: Serum TGF-β1 and CD14 predicts response to anti-TNF-α therapy in IBD

**Journal name**: Journal of Immunology Research

Stepan Coufal^1^ª, Miloslav Kverka^1^ª, Jakub Kreisinger^3^, Tomas Thon^1^, Filip Rob^2^, Martin Kolar^5^, Zuzana Reiss^1^, Dagmar Schierova^1^, Klara Kostovcikova^1^, Radka Roubalova^1^, Lukas Bajer^1,4^, Zuzana Jackova^1^, Martin Mihula^1^, Pavel Drastich^4^, Jana Tresnak Hercogova^2,7^, Michaela Novakova^2^, Martin Vasatko^5^, Milan Lukas^5,6^, Helena Tlaskalova-Hogenova^1^ and Zuzana Jiraskova Zakostelska^1^*

^1^Laboratory of cellular and molecular immunology, Institute of Microbiology of the Czech Academy of Sciences, Czech Academy of Sciences, Prague, Czech Republic

^2^Second Faculty of Medicine, University Hospital Bulovka, Dermatovenerology Department, Charles University, Prague, Czech Republic

^3^Laboratory of Animal Evolutionary Biology, Faculty of Science, Charles University, Department of Zoology, Prague, Czech Republic

^4^Institute for Clinical and Experimental Medicine, Hepatogastroenterology Department, Prague, Czech Republic

^5^ISCARE a.s., IBD Clinical and Research Centre, Prague, Czech Republic

^6^Institute of Medical Biochemistry and Laboratory Diagnostics, General University Hospital and First Faculty of Medicine, Charles University, Prague, Czech Republic

^7^Prof. Hercogova Dermatology, Prague

ªThese authors contributed equally

*** Correspondence:**

Zuzana Jiraskova Zakostelska
[zakostelska@biomed.cas.cz](mailto:zakostelska@biomed.cas.cz)

**Supplementary table 1: Summary of anthropometric and clinical parameters from validation cohort composed from patients with IBD undergoing long-term successful anti-TNF-α therapy and healthy individuals.**

| **Validation cohort** | | | |
| --- | --- | --- | --- |
| **Group** | **CD (N=41)** | **UC (N=16)** | **HC (N=46)** |
| **Age (years)** | 40  (32; 49)  (**a**) | 43  (36; 53)  (**a**) | 35  (27;43)  (**a**) |
| **Gender (F, N)** | 20  (**a**) | 4  (**a**) | 25  (**a**) |
| **BMI** | 25.6  (23.3; 29.4)  (**a**) | 26.0  (22.7; 28.8)  (**a**) | 24.0  (20.9; 27.2)  (**a**) |
| **Smoking (N)** | 17  (**a**) | 1  (**b**) | 10  (**ab**) |
| **Infliximab/Adalimumab (N)** | 30/11  (**a**) | 14/2  (**a**) | - |
| **CRP (mg/L)** | 2.9  (1.3; 6.1)  (**a**) | 2.4  (1.3; 3.2)  (**ab**) | 1.1  (0.5; 2.6)  (**b**) |
| **WBC** | 7.2  (5.9; 8.9)  (**a**) | 7.4  (5.7; 9.4)  (**a**) | 5.5  (4.8; 6.9)  (**b**) |
| **PLT** | 263.5  (209.5; 304.3)  (**a**) | 261.5  (236.5; 301.5)  (**a**) | 250.0  (218.0; 292.0)  (**a**) |
| **Hb (µg/L)** | 144.5  (139.0; 152.8)  (**a**) | 151.5  (131.3; 156.0)  (**a**) | 139.0  (132.0; 155.0)  (**a**) |
| **FC (µg/g)** | 192.0  (81.0; 940.0)  (**a**) | 272.0  (138.0; 1535.0)  (**a**) | - |
| **Clinical score (HBI)** | 1  (0; 5) | - | - |
| **Clinical score (pMAYO)** | - | 1  (0.3; 3) | - |

_In total, we collected serum samples from 46 healthy individuals, 30 CD patients with infliximab treatment, 11 CD patients with adalimumab treatment, 14 UC patients with infliximab, 2 UC patients with adalimumab. Medians are reported with the first and third quartiles in parentheses. N (number of participants), CD (Crohn’s disease), UC (ulcerative colitis), HBI (Harvey-Bradshaw Index), pMAYO (partial Mayo score), CRP (C-reactive protein), FC (fecal calprotectin), Hb (hemoglobin), PLT (platelet count), WBC (white blood cells). Statistical differences between groups are depicted by letters at the level of p<0.05. When same letter is present (e.g. a, a) in one row of the table, no statistical difference between group or time points is present. When two different letters are present (e.g. a, b) in the one row of the table, there is statistical difference between these observations at the level of p<0.05._

**Supplementary table 2: The model performance information described by area under curve (AUC) and Akaike Information Criterion (AIC).**

| Model | AIC | Delta_AIC | AUC of model |
| --- | --- | --- | --- |
| TGF-β1+CD14+MMP9+MBL+LBP+L-FABP | 14.08 | 0.0 | 1.0 |
| TGF-β1+CD14+MMP9+MBL+LBP | 28.24 | 14.16 | 0.955 |
| TGF-β1+CD14+MMP9+MBL | 29.24 | 15.16 | 0.955 |
| TGF-β1+CD14+MMP9 | 28.96 | 14.88 | 0.938 |
| TGF-β1+CD14 | 29.37 | 15.29 | 0.936 |
| TGF-β1 | 29.02 | 14.94 | 0.898 |


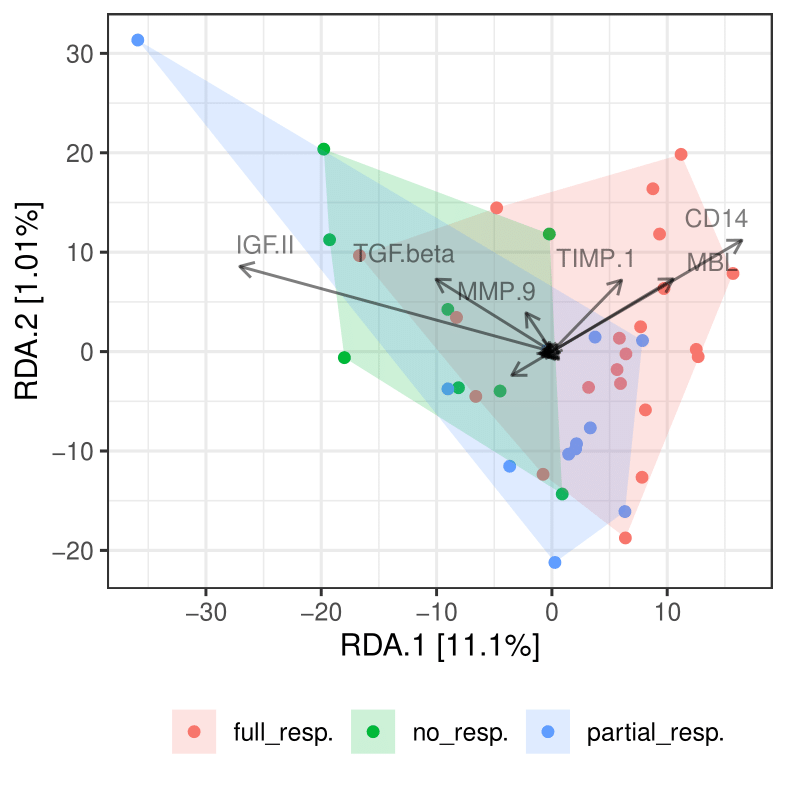


**Supplementary fig. 1 Rda ordination.** Rda biplot shows sample ordination along

the two constrained RDA axes. Ordination space occupied by each sample group is highlighted by convex Hull polygons.

**
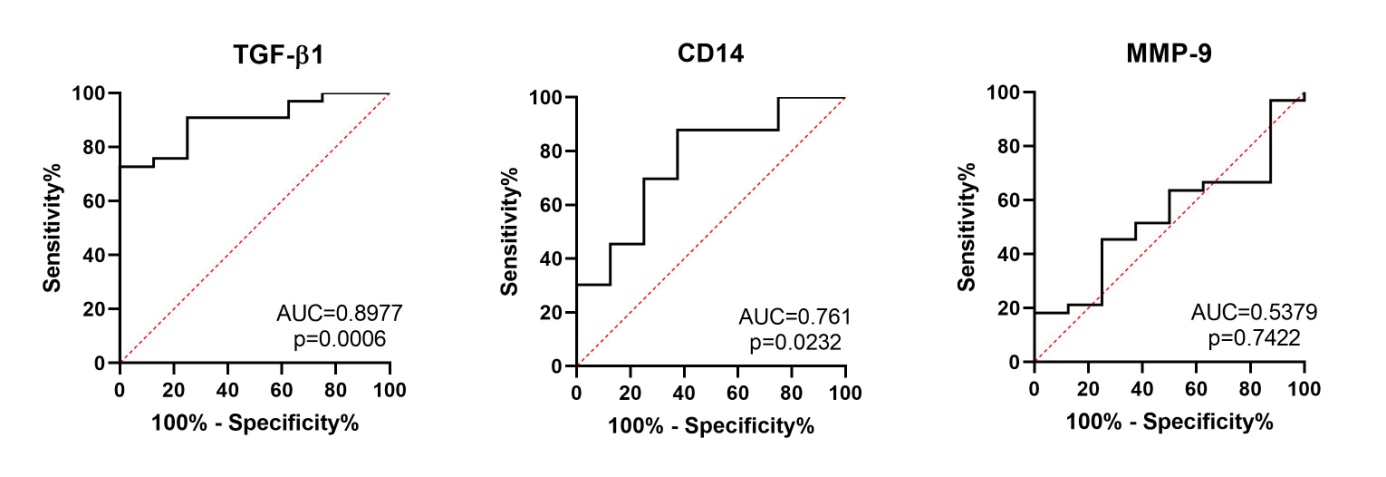
**

**
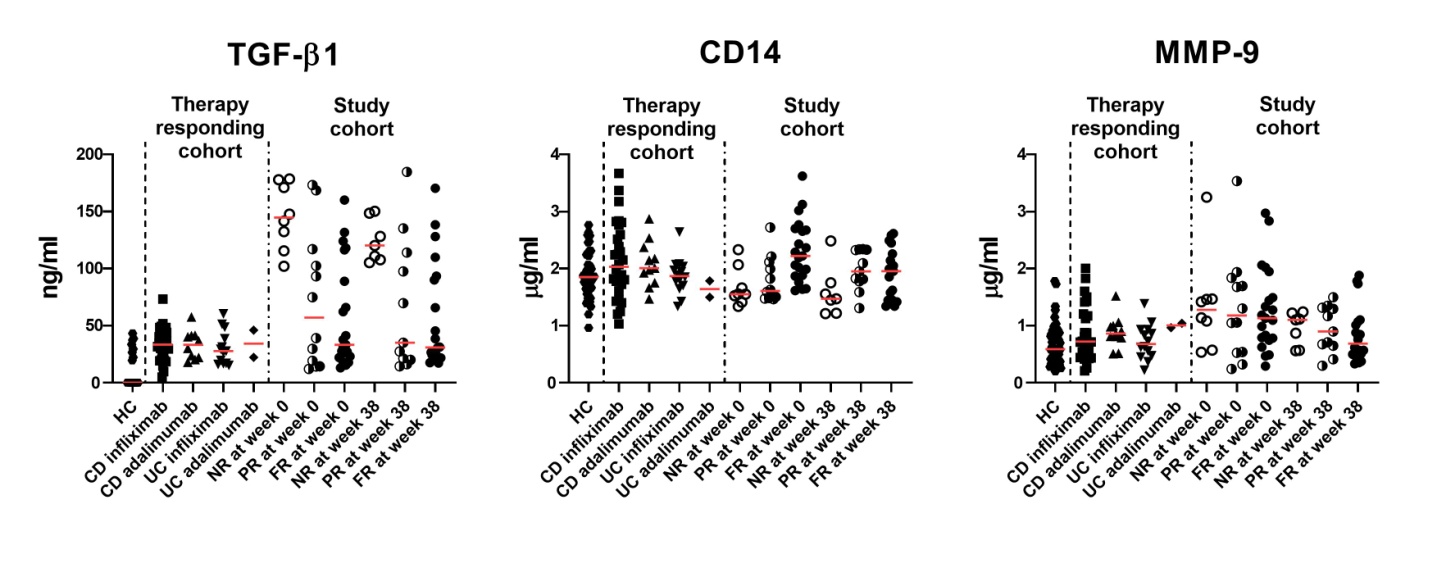
Supplementary fig. 2 The ROC analysis of TGF-β1, CD14 and MMP-9.** Analysis of ROC for individual markers with calculated area under curve (AUC) (* p < 0.05; *** p < 0.001).

**Supplementary fig. 3 The analyses of serum TGF-β1, CD14 and MMP-9 in therapy responding cohorts.** Analysis of the level of three main molecules based on our model in sera of IBD patients (CD, UC) with long-term anti-TNF-α therapy (infliximab or adalimumab) and healthy individuals (HC)(median is depicted as a red line).
